# Neuropathologic and Clinical Findings in Young Contact Sport Athletes Exposed to Repetitive Head Impacts

**DOI:** 10.1001/jamaneurol.2023.2907

**Published:** 2023-08-28

**Authors:** Ann C. McKee, Jesse Mez, Bobak Abdolmohammadi, Morgane Butler, Bertrand Russell Huber, Madeline Uretsky, Katharine Babcock, Jonathan D. Cherry, Victor E. Alvarez, Brett Martin, Yorghos Tripodis, Joseph N. Palmisano, Kerry A. Cormier, Caroline A. Kubilus, Raymond Nicks, Daniel Kirsch, Ian Mahar, Lisa McHale, Christopher Nowinski, Robert C. Cantu, Robert A. Stern, Daniel Daneshvar, Lee E. Goldstein, Douglas I. Katz, Neil W. Kowall, Brigid Dwyer, Thor D. Stein, Michael L. Alosco

**Affiliations:** 1 Veterans Affairs (VA) Boston Healthcare System, US Department of Veteran Affairs, Boston, Massachusetts; 2 Alzheimer’s Disease Research Center and Chronic Traumatic Encephalopathy Center, Chobanian and Avedisian School of Medicine, Boston University, Boston, Massachusetts; 3 Department of Neurology, Chobanian and Avedisian School of Medicine, Boston University, Boston, Massachusetts; 4 Department of Pathology and Laboratory Medicine, Chobanian and Avedisian School of Medicine, Boston University, Boston, Massachusetts; 5 VA Bedford Healthcare System, US Department of Veteran Affairs, Bedford, Massachusetts; 6 National Center for PTSD, VA Boston Healthcare, Boston, Massachusetts; 7 Department of Biostatistics, Boston University School of Public Health, Boston, Massachusetts; 8 Department of Anatomy and Neurobiology, Chobanian and Avedisian School of Medicine, Boston University, Boston, Massachusetts; 9 Biostatistics and Epidemiology Data Analytics Center, Boston University School of Public Health, Boston, Massachusetts; 10 Concussion Legacy Foundation, Boston, Massachusetts; 11 Department of Neurosurgery, Emerson Hospital, Concord, Massachusetts; 12 Department of Neurosurgery, Chobanian and Avedisian School of Medicine, Boston University, Boston, Massachusetts; 13 Department of Rehabilitation Medicine, Harvard Medical School, Boston, Massachusetts; 14 Department of Psychiatry, Chobanian and Avedisian School of Medicine, Boston University, Boston, Massachusetts; 15 Department of Biomedical, Electrical, and Computer Engineering, Boston University College of Engineering, Boston, Massachusetts; 16 Braintree Rehabilitation Hospital, Braintree, Massachusetts

## Abstract

**Importance:**

Young contact sport athletes may be at risk for long-term neuropathologic disorders, including chronic traumatic encephalopathy (CTE).

**Objective:**

To characterize the neuropathologic and clinical symptoms of young brain donors who were contact sport athletes.

**Design, Setting, and Participants:**

This case series analyzes findings from 152 of 156 brain donors younger than 30 years identified through the Understanding Neurologic Injury and Traumatic Encephalopathy (UNITE) Brain Bank who donated their brains from February 1, 2008, to September 31, 2022. Neuropathologic evaluations, retrospective telephone clinical assessments, and online questionnaires with informants were performed blinded. Data analysis was conducted between August 2021 and June 2023.

**Exposures:**

Repetitive head impacts from contact sports.

**Main Outcomes and Measures:**

Gross and microscopic neuropathologic assessment, including diagnosis of CTE, based on defined diagnostic criteria; and informant-reported athletic history and informant-completed scales that assess cognitive symptoms, mood disturbances, and neurobehavioral dysregulation.

**Results:**

Among the 152 deceased contact sports participants (mean [SD] age, 22.97 [4.31] years; 141 [92.8%] male) included in the study, CTE was diagnosed in 63 (41.4%; median [IQR] age, 26 [24-27] years). Of the 63 brain donors diagnosed with CTE, 60 (95.2%) were diagnosed with mild CTE (stages I or II). Brain donors who had CTE were more likely to be older (mean difference, 3.92 years; 95% CI, 2.74-5.10 years) Of the 63 athletes with CTE, 45 (71.4%) were men who played amateur sports, including American football, ice hockey, soccer, rugby, and wrestling; 1 woman with CTE played collegiate soccer. For those who played football, duration of playing career was significantly longer in those with vs without CTE (mean difference, 2.81 years; 95% CI, 1.15-4.48 years). Athletes with CTE had more ventricular dilatation, cavum septum pellucidum, thalamic notching, and perivascular pigment-laden macrophages in the frontal white matter than those without CTE. Cognitive and neurobehavioral symptoms were frequent among all brain donors. Suicide was the most common cause of death, followed by unintentional overdose; there were no differences in cause of death or clinical symptoms based on CTE status.

**Conclusions and Relevance:**

This case series found that young brain donors exposed to repetitive head impacts were highly symptomatic regardless of CTE status, and the causes of symptoms in this sample are likely multifactorial. Future studies that include young brain donors unexposed to repetitive head impacts are needed to clarify the association among exposure, white matter and microvascular pathologic findings, CTE, and clinical symptoms.

## Key Points

**Question:**

What are the neuropathologic and clinical findings in a convenience sample of young, deceased, symptomatic contact sport athletes?

**Findings:**

In this case series of 152 contact sport athletes younger than 30 years at the time of death, chronic traumatic encephalopathy (CTE) was found in 63 (41.4%), with nearly all having mild CTE (stages I and II). Neuropathologic abnormalities associated with CTE included ventricular enlargement, cavum septum pellucidum, thalamic notching, and perivascular pigment–laden macrophage deposition in the frontal white matter.

**Meaning:**

These findings confirm that CTE and other brain pathologies can be found in young, symptomatic contact sport athletes, but the clinical correlates of these pathologic conditions are uncertain.

## Teaser

This cases series describes the neuropathologic and clinical features of young contact sport athletes who were younger than 30 years at the time of death.

## Introduction

Across the world, millions of people are exposed to repetitive head impacts (RHIs) through participation in contact and collision sports, military service, physical violence, and many other activities.^1,2,3,4,5,6^ Repetitive head impacts can result in symptomatic concussions and the much more frequent, nonconcussive injuries that are asymptomatic.^7^ Sustained exposure to RHIs can produce persistent cognitive and neuropsychiatric symptoms^8,9,10,11^ and a progressive, tau-based neurodegenerative disease, chronic traumatic encephalopathy (CTE).^12,13,14,15,16,17,18,19,20,21^ Multiple studies^13,15,22^ link a longer duration of RHI exposure in US football players with increased odds for the presence of CTE and increased severity of CTE. In older American football players with pathologically diagnosed CTE, RHI exposure is also associated with white matter rarefaction,^23,24,25^ loss of myelin associated proteins,^26^ and oligodendrocyte loss.^27^ Emerging data show structural white matter alterations on magnetic resonance imaging (MRI) in young, active, and recently retired contact sport players exposed to RHI,^1,2,28,29,30^ although the pathologic condition underlying these changes is unclear.

A definitive diagnosis of CTE requires neuropathologic evidence of perivascular hyperphosphorylated tau (p-tau) aggregates in neurons, with or without astrocytes, typically at the depths of the sulci in the cerebral cortex.^31,32^ The clinical syndrome associated with CTE is known as traumatic encephalopathy syndrome (TES).^8,33^ On the basis of the National Institute of Neurological Disorders and Stroke (NINDS) consensus diagnostic criteria for TES,^8^ the core clinical features of TES include cognitive impairment, especially episodic memory and executive dysfunction, and neurobehavioral dysregulation, such as impulsivity, explosivity, and emotional dysregulation.^8^ Supportive features include delayed onset (ie, core clinical features starting years after RHI exposure ends), parkinsonism, other motor signs (including amyotrophic lateral sclerosis), depression, anxiety, apathy, and paranoia.

The Understanding Neurologic Injury and Traumatic Encephalopathy (UNITE) Brain Bank^1^ has harvested brains from more than 1350 donors exposed to RHIs who are considered at risk for CTE. Brain donors in the UNITE bank vary widely in age at death and include teenagers and young adults. Chronic traumatic encephalopathy has been reported in individuals as young as 17 years,^13^ yet, to date, there have been no large-scale neuropathologic and clinical studies of young individuals exposed to RHIs. Attention to this age group has several important implications. The study of young athletes allows insight into the earliest features of RHI-induced neuropathologic injury and CTE. Furthermore, it allows analysis in the absence of common age-associated comorbidities. Moreover, most of these young contact sport athletes played only at amateur levels, as part of teams affiliated with educational institutions; consequently, the study of young athletes adds to our understanding of the long-term consequences of amateur contact sports participation. In this report, we describe the neuropathologic and clinical features of 152 brain donors from the UNITE brain bank who were younger than 30 years at the time of death.

## Methods

### Brain Donors and Study Design

The initial sample for this case series included 156 deceased individuals with a history of exposure to RHIs from contact sports participation who donated their brain to the UNITE Brain Bank from February 1, 2008, to September 31, 2022, and were younger than 30 years at the time of death. Race was determined by next-of-kin report and was included to understand the representativeness of the sample and the associated generalizability of the results. Procedures of brain donation have been previously described.^14,34^ The inclusion criterion was based on the presence of a history of exposure to RHIs without regard to symptom status. The restriction to brain donors younger than 30 years was selected to minimize any contribution from age-related conditions. Donors were excluded for poor tissue quality. Four donors were excluded because of incomplete brain fragments or prolonged premortem hypoxia, resulting in a final sample size of 152. Institutional review board approval for brain donation, postmortem clinical record review, interviews with informants, and neuropathologic evaluation was obtained through the Boston University Medical Campus and the Veterans Affairs Bedford Institutional Review Board. The next of kin or legally authorized representative of each brain donor provided written informed consent. The methods for this report followed the appropriate use and reporting of uncontrolled case series in the medical literature reporting guidelines.^35^

### Neuropathologic Evaluation

Neuropathologic evaluation occurred blinded to the clinical evaluation by neuropathologists (A.C.M., B.R.H., V.E.A., and T.D.S.). Pathologic processing and evaluation were conducted using previously published methods and as described in the eAppendix in Supplement 1.^12,13,14,34,36^ Neuropathologic diagnoses were made using NINDS National Institute of Biomedical Imaging and Bioengineering criteria for CTE^31,32^ and well-established criteria for other neurodegenerative diseases.^37,38,39,40^ The CTE p-tau pathologic findings were classified into 4 stages using the McKee staging scheme for CTE.^13,41^ Brain tissue was also evaluated for the presence of vascular pathologic findings, including gross infarcts, microinfarcts, atherosclerosis, and arteriolosclerosis, as well as white matter rarefaction and the presence of perivascular pigment–laden macrophages within the deep cerebral white matter. For pathologic findings rated on a none, mild, or moderate-severe scale (cerebral amyloid angiopathy, white matter rarefaction, atherosclerosis, and arteriolosclerosis), scores were dichotomized as present vs absent. Neuropathologic diagnoses were reviewed by the 4 neuropathologists (A.C.M., B.R.H., V.E.A., and T.D.S.); any discrepancies were resolved by discussion and consensus of the group.

### Clinical Evaluation

Retrospective clinical evaluations with next of kin were performed on 143 brain donors using online surveys and/or structured and semistructured postmortem telephone interviews, as described previously.^34^ Established scales modified for retrospective administration were given to informants of brain donors to assess cognitive symptoms, mood disturbances, and neurobehavioral dysregulation. Scales that assessed cognitive and functional symptoms included BRIEF–A Metacognition Index, Cognitive Difficulties Scale, and the Functional Activities Questionnaire. Scales that assessed neurobehavioral dysregulation included BRIEF–A Behavioral Regulation Index and Barratt Impulsiveness Scale 11. The Apathy Evaluation Scale and Geriatric Depression Scale, 15-item version assess apathy and depression symptoms, respectively. These scales were used as primary outcomes in this study. Further details on clinical protocols are outlined in the eAppendix in Supplement 1. Eighteen brain donors with missing clinical scale data were excluded from clinical analyses.

### Statistical Analysis

Statistical analysis was conducted between August 2021 and June 2023. Qualitative assessments of the neuropathologic and clinical features were performed, and descriptive statistics were generated from SPSS software, version 20 (IBM Inc). Brain donors with and without CTE were compared on demographic, athletic, medical, and sport characteristics using independent-sample *t* tests (for continuous outcomes) and the χ^2^ test or Fisher exact test (for binary outcomes). Neuropathologic and clinical features were compared between brain donors with and without CTE using analysis of covariance for continuous outcomes and binary logistic regression for binary outcomes. The analyses were controlled for age at death. Data were collected and stored using REDCap, version 8.5.1 (Vanderbilt University). A 2-sided *P* < .05 was considered statistically significant.

## Results

Among the 152 brain donors, age at death ranged from 13 to 29 years (mean [SD] age, 22.97 [4.31] years) (Table 1). Eleven brain donors (7.2%) were female and 141 (92.8%) were male; 1 (0.7%) was American Indian or Alaska Native, 27 (17.8%) were Black, 111 (73.0%) were White, and 13 (8.6%) had missing or other race, including multiracial (White–African American, White–Indigenous American, White–Asian Indian, or White–Filipino), Tongan, and unspecified. Race information was not provided on 5 donors. Chronic traumatic encephalopathy was neuropathologically diagnosed in 63 brain donors (41.4%), 1 of whom was a woman (1.6%). Brain donors who had CTE were more likely to be older (mean difference, 3.92 years; 95% CI, 2.74-5.10 years; *P* < .001) and have a reported Black racial identity (16 [25.4%]; *P* = .047). Black brain donors were older than donors of other races (mean difference, 1.61 years; 95% CI, 0.01-3.23 years; *P* = .051) and had more years of football play (mean difference, 3.93 years; 95% CI, 2.07-5.78 years; *P* < .001). Brain donors with CTE had a higher level of education (28 [44.4%] vs 17 [19.1%] donors with a college degree or higher; *P* < .001). Suicide was the most common cause of death, followed by unintentional overdose; there were no differences in cause of death based on CTE status.

**Table 1.** Demographic and Athletic Characteristics of the Brain Donors^a^

Characteristic				*P* value
	Total sample (N = 152)	No CTE (n = 89)	CTE (n = 63)	
Age at death, mean (SD) [range], y	22.97 (4.31) [13-29]	21.35 (4.39) [13-29]	25.27 (2.97) [17-29]	<.001
Sex				
Male	141 (92.8)	79 (88.8)	62 (98.4)	.03
Female	11 (7.2)	10 (11.2)	1 (1.6)	
Racial identity				
American Indian or Alaska Native	1 (0.7)	1 (1.1)	0	.047^b^
Black	27 (17.8)	11 (12.4)	16 (25.4)	
White	111 (73.0)	67 (75.3)	44 (69.8)	
Other or missing^c^	13 (8.6)	10 (11.2)	3 (4.8)	
Educational level				
No or some high school	29 (19.1)	25 (28.1)	4 (6.3)	<.001^d^
High school or GED	20 (13.2)	15 (16.9)	5 (7.9)	
Some college, no degree	52 (34.2)	29 (32.6)	23 (36.5)	
College degree	42 (27.6)	15 (16.9)	27 (42.9)	
More than college or graduate degree	3 (2)	2 (2.2)	1 (1.6)	
Unknown	6 (4)	3 (3.4)	3 (4.8)	
Primary sport played				
US football	92 (60.5)	44 (49.4)	48 (76.2)	<.001^e^
Ice hockey	16 (10.5)	10 (11.2)	6 (9.5)	
Soccer	23 (15.1)	19 (21.4)	4 (6.3)	
Amateur wrestling	9 (5.9)	7 (7.9)	2 (3.2)	
Karate	1 (0.7)	1 (1.1)	0	
Professional wrestling	1 (0.7)	0	1 (1.6)	
Rugby	4 (2.6)	2 (2.2)	2 (3.2)	
Lacrosse	2 (1.3)	2 (2.2)	0	
Equestrian	1 (0.7)	1 (1.1)	0	
Softball	1 (0.7)	1 (1.1)	0	
Ultimate frisbee	1 (0.7)	1 (1.1)	0	
Cycling	1 (0.7)	1 (1.1)	0	
Duration of American football play, mean (SD), y	10.29 (4.19)	8.83 (3.95)	11.64 (3.98)	<.001
Age at first exposure to US football play, mean (SD), y	9.25 (2.71)	9.27 (2.58)	9.23 (2.87)	.94
Highest level of US football played				
Youth	7 (7.6)	5 (11.4)	2 (4.2)	.004^f^
High school	45 (48.9)	31 (7.5)	14 (29.2)	
College	26 (23.3)	5 (11.4)	21 (43.8)	
Semiprofessional	2 (2.2)	2 (4.5)	0	
Professional	12 (13)	1 (2.3)	11 (22.9)	
American football position played				
Offensive lineman	11 (12)	7 (15.9)	4 (8.3)	.20^g^
Tight end	2 (2.2)	1 (2.3)	1 (2.1)	
Quarterback	2 (2.2)	1 (2.3)	1 (2.1)	
Running back	5 (5.4)	1 (2.3)	4 (8.3)	
Wide receiver	4 (4.3)	2 (4.5)	2 (4.2)	
Defensive lineman	8 (8.7)	5 (11.4)	3 (6.3)	
Linebacker	19 (20.7)	5 (11.4)	14 (29.2)	
Defensive back	12 (13)	0	12 (25)	
Punter	0	0	0	
Kicker	0	0	0	
Other	1 (1.1)	0	1 (2.1)	
Multiple	26 (28.3)	20 (45.5)	6 (12.5)	
Unknown	2 (2.2)	2 (4.5)	0	
Traumatic brain injury history				
Concussion count				
Postdefinition, median (range)	11 (0-1000)	10 (1-1000)	15 (0-1000)	.09
Traumatic brain injury with LOC				
Yes	62 (40.8)	38 (42.7)	24 (38.1)	.92
Military history				
Yes	9 (5.9)	6 (6.7)	3 (4.8)	.74
Combat	5 (3.3)	3 (3.4)	2 (3.2)	>.99
Primary cause of death				
Suicide	87 (57.2)	54 (60.7)	33 (52.4)	.25^h^
Unintentional overdose	22 (14.5)	13 (14.6)	9 (14.3)	
Injury	16 (10.5)	7 (7.9)	9 (14.3)	
Cardiovascular disease	4 (2.6)	3 (3.4)	1 (1.6)	
Motor neuron disease	1 (0.7)	0	1 (1.6)	
Neurodegenerative	0	0	0	
Neoplasm	0	0	0	
Other	15 (9.9)	7 (7.9)	8 (12.7)	
Unknown	7 (4.6)	5 (6)	2 (3.2)	

Abbreviations: CTE, chronic traumatic encephalopathy; GED, general educational development; LOC, loss of consciousness; NA, not available.

^a^ Data are presented as number (percentage) of brain donors unless otherwise indicated. Donors with and without CTE were compared using independent-sample *t* tests for continuous measures and the χ^2^ test or Fisher exact test for binary measures. Primary sport play was determined by the sport a donor played for the most years.

^b^ Compared Black vs other.

^c^ Other races include multiracial (White–African American, White–Indigenous American, White–Asian Indian, or White–Filipino), Tongan, and unspecified. Race information was not provided on 5 donors.

^d^ Compared college degree or greater vs others.

^e^ Compared US football vs others.

^f^ Compared professional vs others.

^g^ Compared linemen (offensive and defensive) vs others.

^h^ Compared suicide vs other.

**Figure 1.**
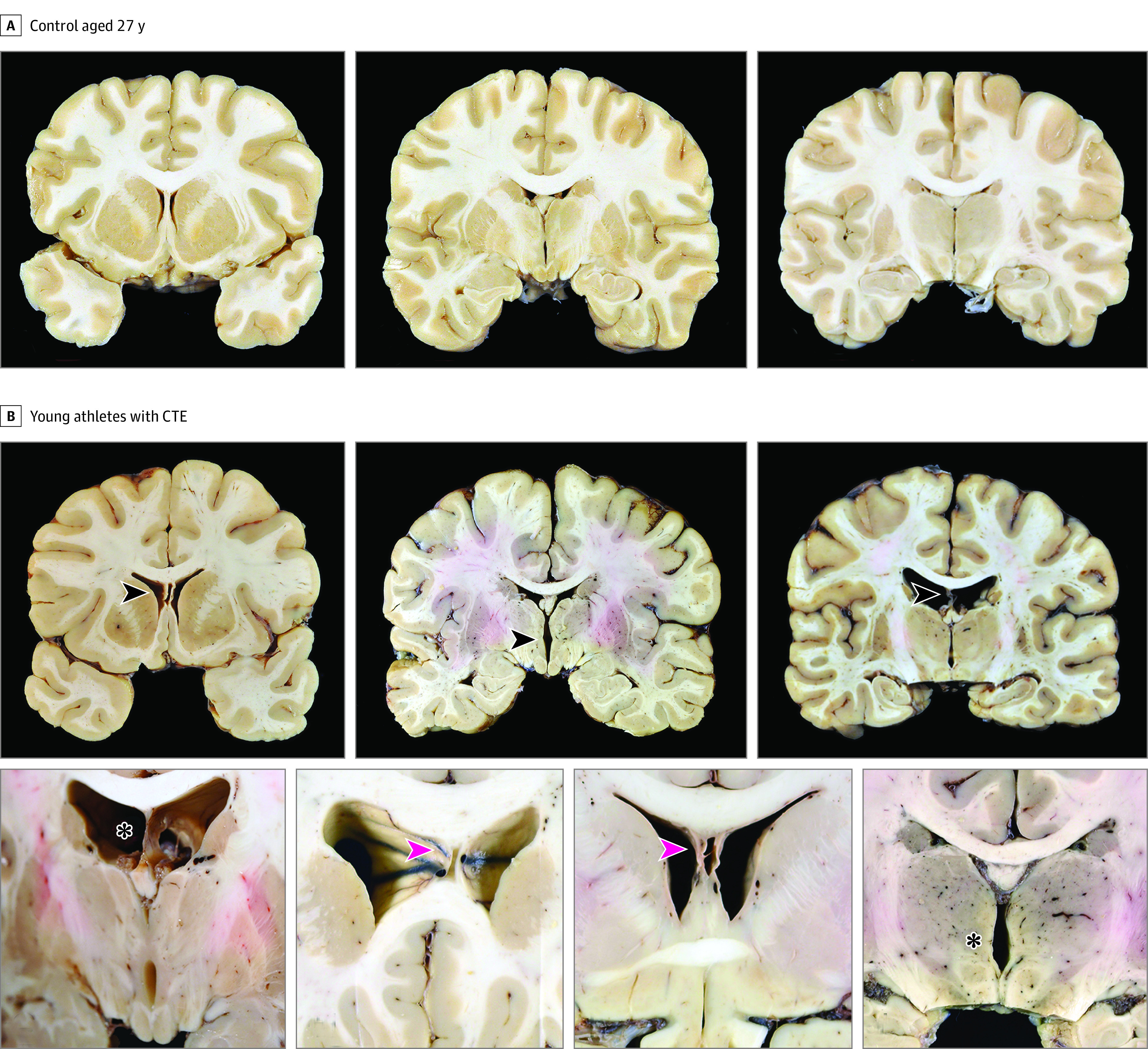
Gross Neuropathologic Features Associated With Chronic Traumatic Encephalopathy (CTE) in Young Athletes A, A 27-year-old control. Coronal brain sections at the level of the caudate, accumbens, and putamen (left); anterior thalamus and mammillary bodies (center); and midthalamus (right). B, Young athletes with CTE. Examples of macroscopic brain abnormalities in CTE. Cavum septum pellucidum (top left; arrowhead), thalamic notch (top center; arrowhead), degeneration of fornix (top right; arrowhead), enlargement of the frontal horns of the lateral ventricles and septal fenestrations (bottom left; asterisk), enlargement of the frontal horns of the lateral ventricles and cavum septum pellucidum (2 bottom center images; arrowheads), and thalamic notch (bottom right; asterisk).

**Figure 2.**
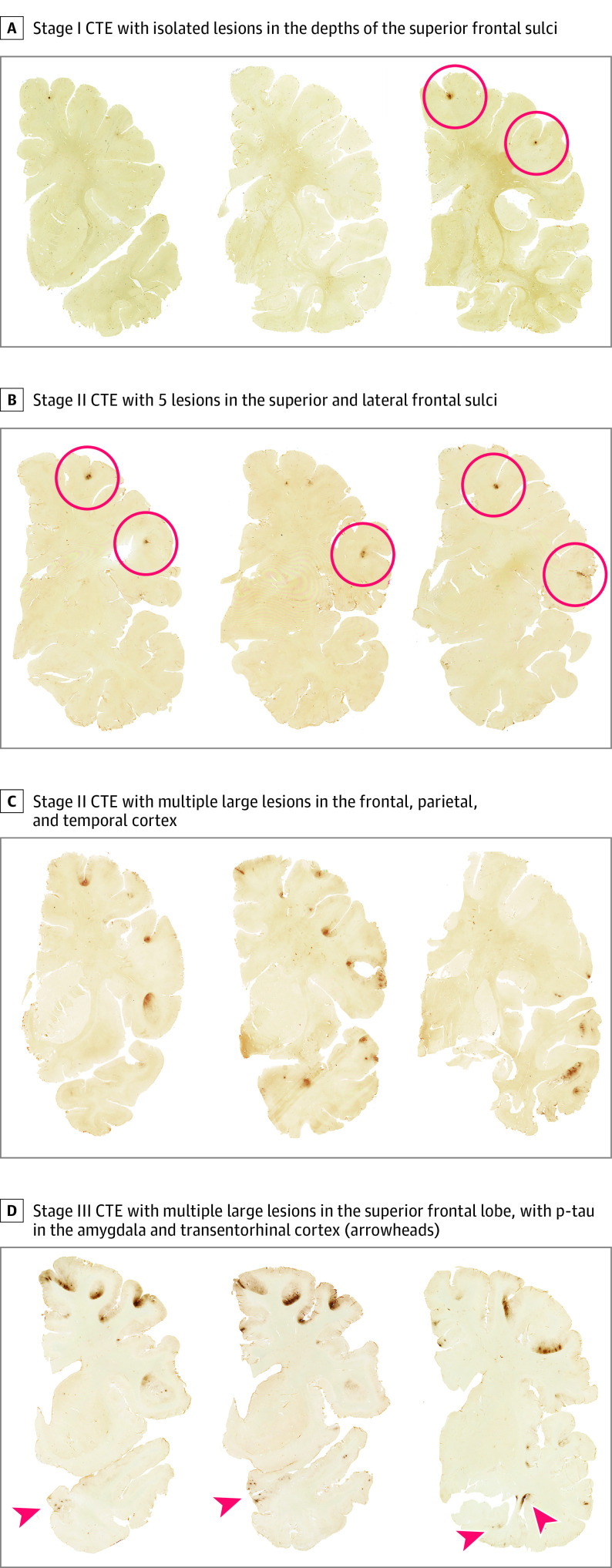
Stages of Chronic Traumatic Encephalopathy (CTE) Severity in Contact Sport Athletes Younger Than 30 Years Hemispheric 50-μm tissue sections immunostained with CP-13, directed against phosphoserine 202 of tau (courtesy of Peter Davies, PhD, Feinstein Institute for Medical Research; 1:200); positive hyperphosphorylated tau (p-tau) immunostaining appears dark brown, showing representative images of CTE in the young athlete brain donors using the McKee staging scheme (I-IV).^1,2^

**Figure 3.**
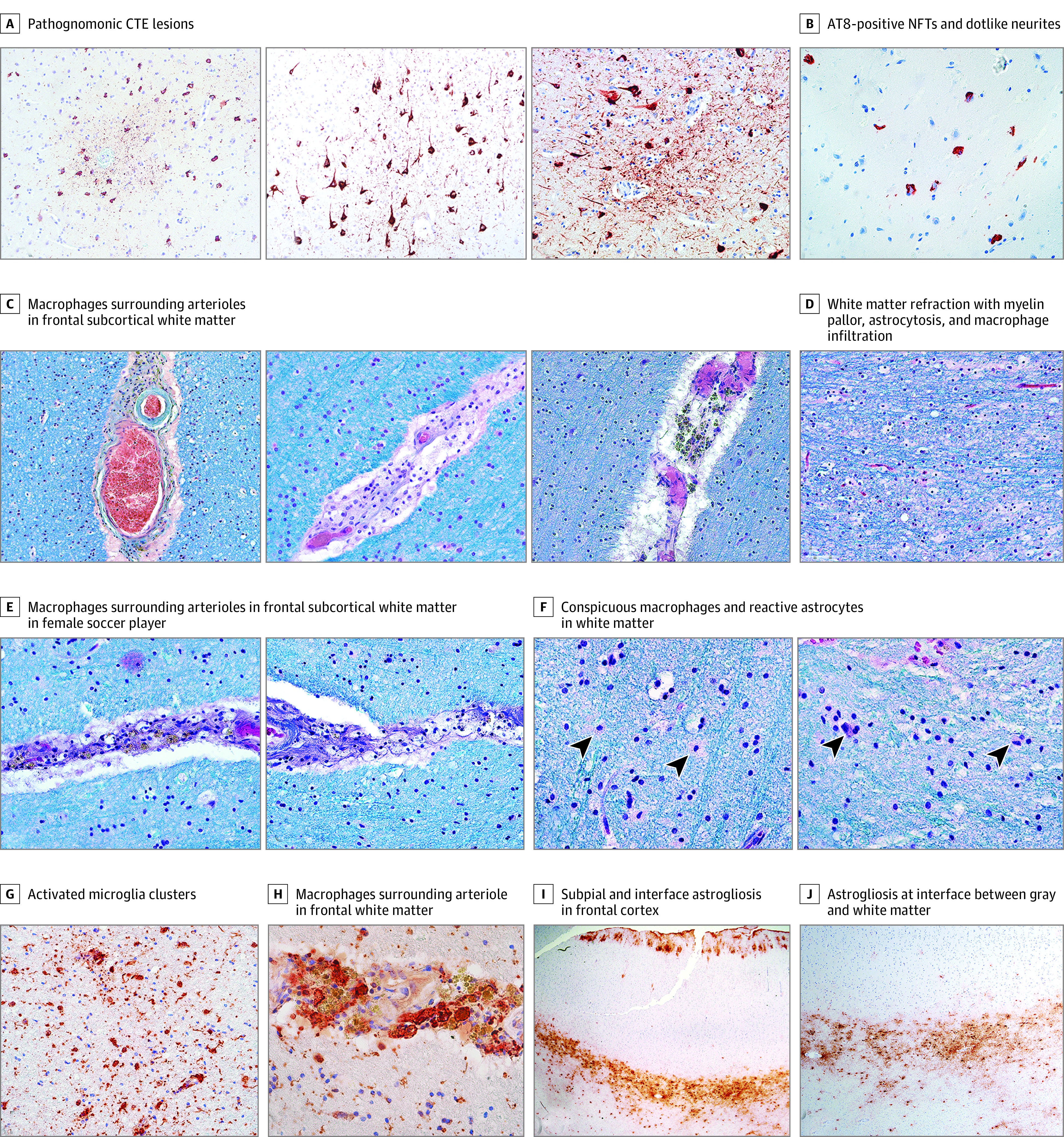
Microscopic Pathologic Features Associated With Chronic Traumatic Encephalopathy (CTE) in Young Athletes A, AT8-immunostained sections of 10-μm formalin-fixed, paraffin-embedded sections shows pathognomonic CTE lesions consisting of hyperphosphorylated tau (p-tau) neurofibrillary tangles (NFTs), pretangles, and dotlike neurites around a central vessel (original magnification ×200). B, Dorsolateral frontal cortex of a 28-year-old female soccer player shows a cluster of AT8-positive NFTs and dotlike neurites at the depth of the sulcus surrounding several small vessels (original magnification ×400). The p-tau lesion measured 1.3 × 1.3 × 1.4 mm in total dimension. C, Dense pigment-laden and clear macrophages surround arterioles in the frontal subcortical white matter (Luxol fast blue, hematoxylin-eosin stain; original magnification ×200). D, White matter rarefaction with myelin pallor, astrocytosis, and macrophage infiltration (Luxol fast blue, hematoxylin-eosin stain; original magnification ×200). E, Dense pigment-laden and clear macrophages surround arterioles in the frontal subcortical white matter in the young female soccer player with CTE (Luxol fast blue, hematoxylin-eosin stain; original magnification ×400). F, Conspicuous macrophages (left, arrowheads) and reactive astrocytes (right, arrowheads) in the white matter in the young female soccer player with CTE (Luxol fast blue, hematoxylin-eosin stain; original magnification ×600). G, Clusters of activated microglia in the frontal white matter of the young female soccer player with CTE (IBA1 immunostaining; original magnification ×600). H, Macrophages surrounding arteriole in the frontal white matter of the young female soccer player with CTE (IBA1 immunostaining; original magnification ×400). I, Subpial and interface astrogliosis in the frontal cortex of the young female soccer player with CTE (glial fibrillary acidic protein immunostaining; original magnification ×20). J, Astrogliosis at the interface between the gray and white matter in the frontal cortex of the young female soccer player with CTE (glial fibrillary acidic protein immunostaining; original magnification ×40).

Of the 63 brain donors diagnosed with CTE, 60 (95.2%) were diagnosed with mild (stages I or II) CTE, including 39 (61.9%) with stage I and 21 (33.3%) with stage II. Three (4.8%) were diagnosed with stage III (Table 2, Figure 1, Figure 2, and Figure 3; eTable 1 in Supplement 1). Those with stage III CTE included 1 former National Football League (NFL) player, 1 college football player, and 1 professional rugby player. No brain donors were diagnosed with stage IV CTE.

**Table 2.** Neuropathologic Characteristics of the Brain Donors^a^

Neuropathologic diagnosis	Total sample (N = 152)	No CTE (n = 89)	CTE (n = 63)	*P* value
CTE				
Stage 0 (no CTE)	89 (58.6)	89 (100)	0	NA
Stage I	39 (25.7)	0	39 (61.9)	
Stage II	21 (13.8)	0	21 (33.3)	
Stage III	3 (2.0)	0	3 (4.8)	
Stage IV	0	0	0	
Gross features				
Brain weight, mean (SD), g	1440.3 (229.71)	1435.61 (221.75)	1447 (242.45)	.77
Cavum septum pellucidum	45 (43.3)	19 (30.2)	26 (63.4)	.001
Septal fenestrations	1 (0.7)	0	1 (2.4)	NA
Frontal atrophy				
None	120 (93)	71 (94.7)	49 (90.7)	.49
Mild	7 (5.4)	3 (4.0)	4 (7.4)	
Moderate-severe	2 (1.6)	1 (1.3)	1 (1.9)	
Temporal atrophy				
None	123 (96.1)	73 (97.3)	50 (94.3)	.65
Mild	5 (3.9)	2 (2.7)	3 (5.7)	
Moderate-severe	0	0	0	
Parietal or occipital atrophy				
None	127 (99.2)	75 (100)	52 (98.1)	.41
Mild	1 (0.8)	0	1 (1.9)	
Moderate-severe	0	0	0	
Hippocampal atrophy				
None	127 (92.7)	77 (93.8)	50 (90.9)	.74
Mild	10 (7.3)	5 (6.2)	5 (9.1)	
Moderate-severe	0	0	0	
Ventricular dilation				
None	75 (71.4)	51 (79.7)	24 (58.5)	.02
Mild	26 (24.8)	12 (18.8)	14 (34.1)	
Moderate-severe	4 (3.9)	1 (1.6)	3 (7.3)	
Thalamic notch	6 (5.5)	1 (1.5)	5 (11.9)	.03
Microscopic features				
White matter rarefaction				
None	78 (52.7)	51 (59.3)	27 (43.5)	.058
Mild	55 (37.2)	28 (32.6)	27 (43.5)	
Moderate-severe	15 (10.2)	7 (8.1)	8 (12.9)	
Frontal perivascular macrophages				
None	28 (20.1)	25 (29.8)	3 (5.5)	<.001
Mild	34 (24.5)	22 (26.2)	12 (21.8)	
Moderate-severe	77 (55.1)	37 (44.1)	40 (72.7)	
Cribriform state				
None	114 (77)	69 (80.2)	45 (72.6)	.28
Mild	18 (12.2)	7 (8.1)	11 (17.7)	
Moderate-severe	16 (10.8)	10 (11.6)	6 (9.7)	
Atherosclerosis				
None	127 (99.2)	77 (98.7)	50 (100)	NA
Mild	1 (0.8)	1 (1.3)	0	
Moderate-severe	0	0	0	
Arteriosclerosis				
None	109 (72.2)	69 (77.5)	40 (64.5)	.08
Mild	29 (19.2)	14 (15.7)	15 (24.2)	
Moderate-severe	13 (8.6)	6 (6.7)	7 (11.3)	
Remote infarcts	0	0	0	NA
Remote microinfarcts	1 (0.7)	0	1 (1.6)	NA
Remote microbleeds	5 (3.3)	3 (3.4)	2 (3.2)	>.99
Diffuse plaques	0	0	0	NA
Neuritic plaques	0	0	0	NA
Cerebral amyloid angiopathy	1 (0.7)	0	1 (1.7)	NA
Lewy bodies	1 (0.7)	0	1 (1.7)	NA
TDP-43	1 (0.7)	0	1 (1.7)	NA
Motor neuron disease	1 (0.7)	0	1 (1.7)	NA

Abbreviations: CTE, chronic traumatic encephalopathy; NA, not available; TDP-43, TAR DNA-binding protein 43.

^a^ Data are presented as number (percentage by category) of brain donors unless otherwise indicated. Some characteristics were not assessed in all participants.

### Football Players

Of the 152 brain donors, 92 (60.5%) played US football as their primary sport, and 48 donors (76.2%) with CTE played football compared with 44 (49.4%) without CTE (*P* < .001). Although significantly more donors with CTE played football at the professional level (11 [22.9%]) compared with those without CTE (1 [2.3%]) (*P* < .001), 37 (58.7%) of those with CTE played football as their primary sport at the amateur level, with 21 (33.3%) playing in college and 16 (25.4%) never playing after high school. Two individuals (3.2%) who played only youth-level football were diagnosed with CTE; however, both had substantial RHI exposure through nonfootball activities, including military service with blast injury (n = 1) and motocross (n = 1). Eleven of 12 professional football players (91.7%) were diagnosed with CTE, including 11 of 11 NFL players (100%). For those who played football, duration of playing career was significantly longer in those with CTE compared with those without CTE (mean difference, 2.81 years; 95% CI, 1.15-4.48 years; *P* < .001). Playing position did not significantly differ between the groups.

### Ice Hockey Players

Of the 16 primary ice hockey players, 6 (37.5%) were diagnosed with CTE. Four amateur ice hockey players were diagnosed with stage I or II CTE; 1 played hockey at the youth level, 2 reached the junior level, and 1 played in college. One of the high school hockey players also played 2 years of college football. One (of 1) National Hockey League (NHL) player had stage II CTE. One (of 1) non-NHL professional hockey player had stage I CTE.

### Soccer and Rugby Players and Wrestlers

Four of 23 athletes (17.4%) who played soccer were diagnosed with CTE. Two high school soccer players were diagnosed with stage I CTE, 1 female collegiate player was diagnosed with stage I CTE, and 1 semiprofessional player was diagnosed with stage II CTE and amyotrophic lateral sclerosis. Two of 9 amateur wrestlers (22.2%) were diagnosed with CTE, 1 who wrestled in high school and 1 in college. There were 4 young rugby players in the sample, including a 17-year-old girl who died of second impact syndrome. Of the 4 players, 2 (50.0%) had CTE, a professional rugby player and a high school rugby player who also played high school football. One (of 1) professional wrestler was diagnosed with stage II CTE.

### Female Brain Donors

There were 11 female brain donors (7.2%). Overall, the female donors were younger (mean [SD] age at death, 20.82 [4.49] years; age range, 16-28 years) and had fewer years of contact sport exposure than the male donors. Their primary sources of exposure to RHIs included college soccer (n = 2), high school soccer (n = 4), high school rugby (n = 1), high school ice hockey (n = 1), professional cycling (n = 1), high school softball (n = 1), and horseback riding (n = 1). The professional cyclist additionally played soccer for 10 years as a winger through high school, and the softball player also played field hockey as a forward through high school. The 1 female player diagnosed with CTE was 28 years old at death and played soccer as a forward for 18 years, beginning at age 3 years and playing through 3 years of Division I collegiate soccer. She was diagnosed with attention-deficit/hyperactivity disorder in college and prescribed stimulants. In addition to 2 concussions without loss of consciousness playing soccer, at age 24 years, she experienced a syncopal episode and a traumatic brain injury with loss of consciousness for 3 minutes. Computed tomographic findings were unremarkable. Four years later, she developed paranoia and suicidal thoughts. At 28 years of age, she died by suicide. Postmortem examination revealed stage I CTE, mild arteriolosclerosis, moderate white matter rarefaction, and marked perivascular pigment–laden macrophages in the frontal white matter (Figure 3).

### Amateur Athletes

Overall, amateur (ie, not semiprofessional or professional) athletes made up 128 of the 152 brain donors (84.2%). Of the 128 amateurs, 45 (35.2%) had CTE; of the 63 donors with CTE, 45 (71.4%) were amateurs. Amateur athletes included football players (78 [60.9%]), soccer players (22 [17.2%]), hockey players (10 [7.8%]), and wrestlers (9 [7.0%]).

### Neuropathologic Features

Among those with available data, cavum septum pellucidum (26 [63.4%] vs 19 [30.2%]; *P* < .001), enlargement of the frontal horns of the lateral ventricles (17 [41.4%] vs 13 [20.4%]; *P* = .02), and a thalamic notch (5 [11.9%] vs 1 [1.5%]; *P* = .03) were significantly more frequent in those with vs without CTE, respectively (Figure 1 and Table 2). In cases of CTE, cavum septum pellucidum was often accompanied by thinning and atrophy of the fornices; 1 case had large septal fenestrations (Figure 1).

### Microscopic Features

In the 63 brain donors diagnosed with CTE, pathognomonic lesions of CTE consisting of p-tau immunoreactive neurofibrillary tangles (NFTs), pretangles, and dotlike neurites surrounding a central blood vessel were found most frequently at the depths of the sulci in the dorsolateral frontal, superior frontal, and superior temporal cortices, followed by the inferior parietal, inferior frontal, and Rolandic cortices (Table 2 and Figures 2 and 3; eTable 1 in Supplement 1). Neurofibrillary tangles were also common in entorhinal cortex, amygdala, and locus coeruleus in those with stage II or III CTE. Multiplex immunofluorescent labeling of the pathognomonic CTE lesions indicated that the NFT-containing cells colocalized with MAP2, a marker of neurons, and that the predominant p-tau isoform was 4 repeat (4R) p-tau (eFigure in Supplement 1). No p-tau–containing astrocytes were found in the pathognomonic CTE lesions or the surrounding neuropil, and no p-tau thorn-shaped astrocytes were found in the subpial region of any brain donor. In all brain regions sampled except the mammillary bodies and calcarine cortex, there were significantly more p-tau NFTs in brain donors with CTE compared with those without (eTable 1 in Supplement 1).

### Non–p-Tau Pathologic Alterations

White matter rarefaction, evident as reduced density of myelinated nerve fibers, conspicuous reactive astrocytes, and macrophages in the white matter, was found more often in those with CTE than those without CTE, although this finding did not reach significance (Figure 3). Perivascular pigment–laden macrophages in the frontal subcortical white matter were found significantly more often in those with CTE (52 [94.5%] vs 59 [70.3%]; *P* < .001) compared with those without CTE (Figure 3 and Table 2).

Among the 92 football players in the sample, years of football play was significantly associated with CTE status (odds ratio, 1.20; 95% CI, 1.07-1.34; *P* = .002) and perivascular macrophages in the frontal white matter (odds ratio, 1.26; 95% CI, 1.08-1.47; *P* = .003). Years of play was not associated with ventricular enlargement, cavum septum pellucidum, thalamic notch, or white matter rarefaction. One 28-year-old brain donor with stage II CTE showed mild cerebral amyloid angiopathy in the frontal leptomeninges, as reported previously.^42^ Neither diffuse nor neuritic plaques were found throughout the sample. One 27-year-old brain donor with CTE stage II had sparse Lewy bodies in the medulla. A semiprofessional soccer player was diagnosed with comorbid stage II CTE and amyotrophic lateral sclerosis.^43^

### Clinical Features

Across the sample of symptomatic brain donors exposed to RHIs, with and without CTE, cognitive, behavioral, and mood symptoms, as reported by informants using standardized scales, were highly frequent (eTable 2 in Supplement 1). There were no statistically significant differences between donors with a CTE diagnosis compared with those without CTE for any clinical symptom (eTable 2 in Supplement 2). Mood and neurobehavioral dysregulation symptoms were particularly frequent. Clinically meaningful symptoms of neurobehavioral dysregulation, based on the BRIEF–A Behavioral Regulation Index, were present in 50 of 88 (56.8%). Mean scores on the Barratt Impulsiveness Scale 11 were also high (mean [SD], 74.25 [16.40]). Clinically meaningful symptoms of apathy (Apathy Evaluation Scale) were present in 72 of 101 donors (71.3%), and clinically meaningful symptoms of depression (Geriatric Depression Scale, 15-item version) were present in 77 of 110 donors (70.0%).

Clinically meaningful symptoms of executive dysfunction, based on the BRIEF–A Metacognition Index, were present in 48 of 88 donors (54.5%). Attention and memory symptoms were less frequent. Functional difficulties were not common, as evidenced by the low Functional Activities Questionnaire scores.

Thirty-four of 119 donors (28.6%) sought substance use treatment during life. Alcohol abuse was present in 60 of 140 (42.9%), drug abuse was present in 54 of 141 (38.3%), and steroid use was present in 7 of 141 (5.0%). Stimulant use was present in 22 of 127 (17.3%). There were no differences in substance, alcohol, steroid, or stimulant use based on CTE status.

## Discussion

Among 152 brain donors exposed to RHIs who donated their brain to the UNITE Brain Bank and were younger than 30 years at the time of death, 63 (41.4%) had autopsy-confirmed CTE, including a female collegiate soccer player (stage I CTE). Chronic traumatic encephalopathy has been previously diagnosed in women who experienced interpersonal violence and frequent head-banging^44,45,46^ and, most recently, in an ex-professional Australian rules football player. This is, to our knowledge, the first report of CTE in a woman who was an amateur soccer player.^47^ Nearly all (60 [95.2%]) of the 63 young brain donors with CTE were diagnosed with mild CTE (stages I or II), and 39 (61.9%) were diagnosed with stage I CTE. Young athletes with neuropathologically confirmed CTE (n = 63) included American football players, ice hockey players, soccer and rugby players, amateur wrestlers, military veterans, and a professional wrestler. The study highlights that CTE can affect amateur as well as professional contact sports athletes, with 45 (71.4%) of the athletes diagnosed with CTE playing only as high as the high school or college level. Professional players also develop CTE at a young age. In this cohort of players younger than 30 years, 11 of 11 NFL players and 1 of 1 NHL players were diagnosed with CTE.

Not all individuals exposed to RHIs will develop CTE, and 89 donors (58.6%) in this sample did not have CTE. Despite the narrow age range of the sample, brain donors with CTE were older, were more likely to play American football, had longer duration of football play, and were more likely to play at an elite level, in line with previous studies^15,41^ of older players. These findings emphasize the dual roles of age and duration of exposure to RHIs in the development of CTE, even among younger individuals.

In young brain donors, CTE was often accompanied by other pathologic abnormalities. Cavum septum pellucidum, often with degeneration and thinning of the fornices, was present significantly more often in donors with CTE. In addition, there was more enlargement of the frontal horns of the lateral ventricles and notching of the medial thalamus.

Chronic traumatic encephalopathy was evident in young athletes as neuronal p-tau aggregates, including NFTs and dotlike neurites, oriented around a small vessel most often in the superior frontal, dorsolateral frontal, and superior temporal cortices. The predominant p-tau isoform in the CTE lesions was 4R p-tau, consistent with a previous study^48^ showing early 4R p-tau predominance and increasing 3-repeat (3R):4R p-tau ratio with increasing severity of CTE. No p-tau astrocytes were found in the parenchyma or subpial region. The lack of p-tau astrocytes was surprising because subpial p-tau thorn-shaped astrocytes are a frequent finding in older individuals with CTE.^31,32,49,50^ Their absence indicates that p-tau astrocytes are not an early or essential feature of CTE. The lack of p-tau astrocytes in young donors with CTE also supports the second NINDS consensus conference conclusion that the pathognomonic CTE lesion requires p-tau aggregates in neurons.^32^

Perivascular pigment–laden macrophages in the frontal white matter were significantly greater in CTE and associated with duration of exposure to RHIs. This finding suggests that disruption of the blood-brain barrier is increased after RHIs and might play a critical role in CTE pathogenesis. Previous autopsy studies^51,52^ in older individuals with CTE also have shown blood-brain barrier dysfunction and microvascular alterations. The vascular injury–associated markers intercellular adhesion molecule 1, vascular cell adhesion molecule 1, and C-reactive protein were increased in individuals with CTE compared with RHI-exposed and RHI-naive controls.^53^ In addition, intercellular adhesion molecule 1 and C-reactive protein levels increased with RHI exposure duration and were associated with increased microglial inflammation and p-tau pathologic findings.^53^ Microglial inflammation correlates with RHI duration and CTE severity,^49^ and reactive astrocytosis at the gray-white matter interface has been reported after RHI and in CTE.^54,55^ Moreover, these findings are in line with dynamic contrast–enhanced MRI studies^56^ of living American football players showing persistent blood-brain barrier dysfunction and white matter alterations on diffusion tensor imaging.

White matter rarefaction was increased in the deceased young athletes with CTE, albeit of marginal significance. White matter changes are also common in older individuals with autopsy-confirmed CTE.^23,26,27,57^ White matter rarefaction in older brain donors with CTE is directly associated with RHI exposure, CTE status, and dementia.^23^ In addition, a single nuclear RNA sequencing study^27^ found that the number of oligodendrocytes was reduced and altered in relative subtype proportions in the dorsolateral frontal white matter of older athletes with CTE compared with controls. A recent autopsy study^26^ of 205 older male brain donors with and without CTE found decreased myelin-associated proteins in frontal white matter that corresponded to years of exposure and age at first exposure to American football. Using high spatial resolution ex vivo diffusion tensor imaging, researchers have reported alterations in fractional anisotropy in white matter underlying sulci with CTE lesions and microscopic evidence of axonal disruption.^57^ In addition, in vivo MRI studies show alterations of the corpus callosum in former NFL players on diffusion imaging scans^25^ and greater volumes of white matter hyperintensities on antemortem fluid-attenuated inversion recovery MRI^24^ and T1 scans.^58^ These observations suggest that white matter rarefaction, perivascular pigment-laden macrophages, neuroinflammation, and interface astrocytosis might represent key components of RHI-induced brain injury and CTE.^1,2,3,4,5,6,28,30^

Clinical symptoms, as reported retrospectively by next of kin, were common in young donors exposed to RHI with and without CTE. Across the sample, based on modified standardized scales, approximately 50% had clinically meaningful symptoms of executive dysfunction. However, difficulties with instrumental activities of daily living were infrequent. Regarding neuropsychiatric symptoms, nearly 60% had symptoms of behavioral dysregulation as measured by the BRIEF–A Behavioral Regulation Index, and impulse control difficulties were also frequently endorsed. Approximately 70% reported meaningful symptoms of depression and apathy. The fact that there were no significant differences in clinical symptoms between those with CTE and those without may be attributable to selection bias (ie, those who donate are more likely to have symptoms). It may also indicate that retrospective review of symptoms with family members might not be granular enough to detect subtle clinical differences. It further implies that the symptoms are not specific to low-stage CTE. Despite all brain donors being symptomatic, 58.6% of the sample did not have CTE, emphasizing that not all contact sport athletes with symptoms have CTE. Nontau pathologic findings related to RHIs (eg, white matter rarefaction, perivascular macrophage infiltration, neuroinflammation, and astrocytosis) and unrelated to RHIs (eg, environmental stressors, medical history, genetic factors, and mental health issues) likely contribute to the clinical symptoms. Future studies that compare RHI-naive individuals with those with varying severities of RHI exposure, with and without CTE, are required to determine whether p-tau pathologic findings, microvascular alterations, or white matter loss are associated with cognitive, behavioral, or mood alterations that occur after RHIs and in CTE in young people.

There is a clear need for improved clinical characterization of living athletes across the age spectrum who have been exposed to RHIs through prospective objective assessments. The importance of prospective research studies on young athletes is underscored by the difficulty of assessing and managing symptoms after RHI in living athletes and the substantial contribution of uncertainty to their psychological stress. A recent study^59^ reported that one-third of former NFL players are “extremely concerned” about cognitive difficulties and “having CTE.” There is also a critical need for symptomatic individuals exposed to RHIs to be medically evaluated and followed up, because their symptoms can often be successfully treated in a clinical care setting.

### Limitations

This study has some limitations. We did not evaluate the incidence or prevalence of CTE in the general population or in young contact sport athletes and other individuals exposed to RHIs, and no estimates of incidence or prevalence can be implied or concluded from this study. This study is constrained to brain donors whose families desired a neuropathologic examination after their loved one’s death, primarily White male football players. There are limitations of ascertainment bias in studies associated with participation in a brain donation program, and those with symptoms during life, regardless of RHI or CTE status, are more likely to donate, potentially explaining the high rates of symptoms in the overall sample. The study is also limited by the lack of a comparison group that is representative of all young individuals exposed to RHIs and a control group of age- and sex-matched individuals not exposed to RHIs. Notably, such resources (ie, autopsies of young age individuals unexposed to RHIs) are extremely limited, underscoring the novelty and importance of this sample. Future studies comparing RHI-naive with RHI-exposed young brain donors will help isolate the clinical and neuropathologic effects of RHIs independent of CTE.

## Conclusion

In a convenience brain bank sample of 152 young athletes exposed to RHI who were younger than 30 years at the time of death, 63 (41.4%) had neuropathologic evidence of CTE, including 1 female athlete. Most young athletes with CTE played at the high school and college levels, and sports included amateur football, ice hockey, rugby and soccer, and wrestling. Young athletes with CTE had significantly more ventricular dilatation, cavum septum pellucidum, thalamic notching, p-tau pathologic findings, and perivascular pigment–laden macrophages in the frontal white matter than those without CTE. Young donors exposed to RHIs were highly symptomatic regardless of CTE status, and the causes of symptoms in this sample, as reported by informants, are likely multifactorial and include RHI- and non–RHI-related causes. Furthermore, despite all donors being symptomatic, 58.6% did not have pathologic evidence of CTE. Future studies that include young brain donors unexposed to RHIs are needed to clarify the association among RHI exposure, white matter and microvascular pathologic findings, CTE, and clinical symptoms.

## Notes

Supplement 1. **eAppendix.** Supplemental Method **eTable 1.** Semi-Quantitative Ratings of Regional P-tau Severity **eTable 2.** Clinical Scales of Young Brain Donors **eFigure.** Multiplex Immunofluorescent Labeling of the CTE Pathognomonic Lesion

Supplement 2.
Data Sharing Statement
