# Supplementary material for: Neuropathologic and Clinical Findings in Young Contact Sport Athletes Exposed to Repetitive Head Impacts
Source: JAMA Neurol. 2023 Aug 28;80(10):1037–50. doi: 10.1001/jamaneurol.2023.2907 (PMC10463175; doi:10.1001/jamaneurol.2023.2907)
Supplement: Supplement 1. — eAppendix. Supplemental Method eTable 1. Semi-Quantitative Ratings of Regional P-tau Severity eTable 2. Clinical Scales of Young Brain Donors eFigure. Multiplex Immunofluorescent Labeling of the CTE Pathognomonic Lesion [file jamaneurol-e232907-s001.pdf]

Supplementary Online Content

McKee AC, Mez J, Abdolmohammadi B, et al. Neuropathologic and clinical findings in young contact sport athletes exposed to repetitive head impacts. *JAMA Neurol.* Published online August 28, 2023. doi:10.1001/jamaneurol.2023.2907

eAppendix. Supplemental Methods

eTable 1. Semi-Quantitative Ratings of Regional P-tau Severity

eTable 2. Clinical Scales of Young Brain Donors

eFigure. Multiplex Immunofluorescent Labelling of the CTE Pathognomonic Lesion

This supplementary material has been provided by the authors to give readers additional information about their work.

© 2023 McKee AC et al. *JAMA Neurology*.

eAppendix.

Supplemental pathological methods:

Tissue was obtained from the dorsolateral frontal cortex (DLFC) and processed as previously described.[1,2] Multiplex immunofluorescence was performed on 10μm thick sections using the Akoya Bioscience Opal Polaris 7 color manual IHC detection kit per the manufacturer's protocol and as previously described.[1,2] Sections were incubated with antibodies to AT8 (Invitrogen, 1:500), MAP2 (BioLegend, 1:500), 3R (RD3, Millipore Sigma, 1:3000), 4R (ET3, gift from Peter Davies, 1:100), Aquaporin (Aquaporin-4, Millipore, 1:750), IBA1 (Wako, 1:1000), and GFAP (BioLegend, 1:750) and stained with DAPI or hematoxylin to label cell nuclei. Sections were imaged using an Akoya Bioscience Vectra Polaris Digital Slide Scanner. Automated spectral unmixing algorithms were created using inForm software (Akoya Biosciences) and applied to whole-slide scans to remove tissue autofluorescence and spectral overlap.

Supplemental clinical methods:

Potential brain donors were evaluated for eligibility using a standard set of inclusion and exclusion criteria predominantly oriented around contact sport participation, as described previously.[2] Sports considered to be contact sports for men include American football, ice hockey, soccer, amateur and entertainment wrestling, boxing, rugby, lacrosse, martial arts, equestrian, bull riding, ultimate frisbee, and Australian rules football. For women, softball, gymnastics, cheerleading, and field hockey were additionally considered to be contact sports. Primary sport play was defined as the sport a brain donor played for the most years.

Retrospective clinical evaluations with next of kin were performed using online surveys and/or structured and semi-structured post-mortem telephone interviews, as described previously.[1] Researchers were blind to the neuropathological analysis and informants were interviewed before receiving the neuropathological results. The UNITE study has evolved over time with a more standardized, systematic assessment adopted in 2014. After January 2014, we began to implement scales of cognitive and neuropsychiatric symptoms (all modified to be informant-based). The Behavior Rating Inventory of Executive Function-Adult Version (BRIEF-A) Metacognition Index and the Cognitive Difficulties Scale (CDS) were used to assess difficulties with executive function and general cognitive difficulties (with emphasis on attention and memory, respectively). The Functional Activities Questionnaire (FAQ) assessed instrumental activities of daily living. Barratt Impulsiveness Scale (BIS-11) was administered to assess symptoms of impulse control, the Brown-Goodwin Lifetime History of Aggression scale (BGLHA), Geriatric Depression Scale 15-item version (GDS-15) and the Apathy Evaluation Scale (AES) were administered to assess aggression, depression, and apathy, respectively. Only the CDS, FAQ, and GDS-15 were administered pre-2014. All scales were collected via a combination of online surveys and structured telephone interviews.

Demographics, educational attainment, athletic history (type of sports played, level, position, age of first exposure and duration), military history (branch, location of service and duration of combat exposure), and traumatic brain injury (TBI) history (including number of concussions) were queried during a telephone interview (pre-2014) and/or using an online questionnaire (2014 and on). TBI history was queried using informant versions of the Ohio State University TBI Identification Method Short Form and two questionnaires adapted from published studies that address military-related head injuries and concussions.[1]

References

1. Mez J, Daneshvar DH, Kiernan PT, et al. Clinicopathological Evaluation of Chronic Traumatic Encephalopathy in Players of American Football. *JAMA*. 2017;318(4):360-370. doi:10.1001/jama.2017.8334

2. Mez J, Solomon TM, Daneshvar DH, et al. Assessing clinicopathological correlation in chronic traumatic encephalopathy: rationale and methods for the UNITE study. *Alzheimers Res Ther*. 2015;7(1):62. doi:10.1186/s13195-015-0148-8

© 2023 McKee AC et al. *JAMA Neurology*.

eTable 1. Semi-Quantitative Ratings of Regional P-tau Severity

**Semi-quantitative p-tau severity**	**n**	**%**	**n**	**%**	**n**	**%**	
Dorsolateral frontal cortex							
None	90	61.2	78	90.7	12	19.7	<0.001
Mild	30	20.4	8	9.3	22	36	
Moderate-severe	27	18.3	0	0	27	44.3	
Rolandic cortex							
None	121	86.4	84	100	37	66.1	<0.001
Mild	12	8.6	0	0	12	21.4	
Moderate-severe	7	5	0	0	7	12.5	
Inferior frontal cortex							
None	115	82.7	82	98.8	33	58.9	<0.001
Mild	16	11.5	1	1.2	15	26.8	
Moderate-severe	8	5.7	0	0	8	14.3	
Superior frontal cortex							
None	96	70.1	79	96.3	17	30.9	<0.001
Mild	17	12.4	3	3.7	14	25.5	
Moderate-severe	24	17.5	0	0	24	43.6	
Septal cortex							
None	113	87.6	79	100	34	68	<0.001
Mild	8	6.2	0	0	8	16	
Moderate-severe	8	6.2	0	0	8	16	
Insula							
None	121	89	85	100	36	70.6	<0.001
Mild	7	5.1	0	0	7	13.7	
Moderate-severe	8	5.9	0	0	8	15.7	
Inferior parietal cortex							
None	113	80.1	83	100	30	51.7	<0.001
Mild	15	10.6	0	0	15	25.9	
Moderate-severe	13	9.2	0	0	13	22.4	
Superior temporal cortex							
None	115	81	84	98.8	31	54.4	<0.001
Mild	9	6.3	1	1.2	8	14	
Moderate-severe	18	12.7	0	0	18	31.6	
Temporal pole							
None	110	83.3	77	97.5	33	62.3	<0.001
Mild	12	9.1	2	2.5	10	18.9	

© 2023 McKee AC et al. *JAMA Neurology*.

Moderate-severe	10	7.6	0	0	10	18.9	
Calcarine							
None	137	99.3	85	100	52	98.1	0.384
Mild	1	0.7	0	0	1	1.9	
Moderate-severe	0	0	0	0	0	0	
CA1							
None	127	90.1	83	97.6	44	78.6	<0.001
Mild	9	6.4	2	2.4	7	12.5	
Moderate-severe	5	3.5	0	0	5	8.9	
CA2							
None	135	96.4	85	100	50	90.9	0.008
Mild	4	2.9	0	0	4	7.3	
Moderate-severe	1	0.7	0	0	1	1.8	
CA4							
None	132	93.6	85	100	47	83.9	<0.001
Mild	3	2.1	0	0	3	5.4	
Moderate-severe	6	4.2	0	0	6	10.7	
Entorhinal							
None	114	79.7	80	94.1	34	58.6	<0.001
Mild	15	10.5	4	4.7	11	19	
Moderate-severe	14	9.8	1	1.2	13	22.4	
Amygdala							
None	116	84.1	80	96.4	36	65.5	<0.001
Mild	10	7.2	2	2.4	8	14.5	
Moderate-Severe	12	8.7	1	1.2	11	20	
Thalamus							
None	130	90.3	86	100	44	75.9	<0.001
Mild	10	6.9	0	0	10	17.2	
Moderate-Severe	4	2.8	0	0	4	6.9	
Mammillary body							
None	109	96.5	69	100	40	90.9	0.021
Mild	4	3.5	0	0	4	9.1	
Moderate-severe	0	0	0	0	0	0	
Substantia innominata							
None	118	88.7	73	96.3	39	76.5	0.001
Mild	8	6	3	3.7	5	9.8	
Moderate-severe	7	5.3	0	0	7	13.7	
Substantia nigra							

© 2023 McKee AC et al. *JAMA Neurology*.

None	128	92.1	81	98.8	47	82.5	<0.001
Mild	9	6.5	0	0	9	15.8	
Moderate-Severe	2	1.4	1	1.2	1	1.8	
Median raphe nucleus							
None	129	93.5	84	100	45	83.3	<0.001
Mild	8	5.8	0	0	8	14.8	
Moderate-severe	1	0.7	0	0	1	1.9	
Locus coeruleus							
None	86	64.2	67	80.7	19	37.3	<0.001
Mild	34	25.4	15	18.1	19	37.3	
Moderate-severe	14	10.4	1	1.2	13	25.4	

Donors with and without CTE were compared using independent samples t-tests for continuous measures, and chi-square or Fisher's Exact Test for binary measures.

© 2023 McKee AC et al. *JAMA Neurology*.

eTable 2. Clinical Scales of Young Brain Donors

Clinical features	All (%)	No CTE (%)	CTE (%)	p-value
Clinical scales				
BRIEF-A MI T-score, mean (SD) (n=88)	65.7 (15.5)	66.61 (14.9)	64.56 (16.3)	0.540
Impaired (n,%)	48 (54.5)	28 (57.1)	20 (51.3)	0.583
BRIEF-A BRI T-score, mean (SD) (n=88)	66.7 (16.3)	66.43 (16.4)	67.05 (16.3)	0.860
Impaired (n,%)	50 (56.8)	27 (55.1)	23 (59)	0.716
CDS (raw), mean (SD) (n=108)	42.6 (31.3)	42.32 (31.2)	43.02 (31.8)	0.910
FAQ (raw), mean (SD) (n=119)	1.64 (2.7)	1.3 (2.2)	2.1 (3.2)	0.129
Impaired (n,%)	3 (2.5)	1 (1.4)	2 (4)	0.572
AES (raw), mean (SD) (n=101)	41.87 (13.3)	43.48 (12.8)	39.21 (13.8)	0.119
Impaired (n,%)	72 (71.3)	49 (77.8)	23 (62.2)	0.063
GDS-15 (raw), mean (SD) (n=110)	8.39 (4.9)	8.57 (4.9)	8.12 (5.1)	0.643
Impaired (n,%)	77 (70)	50 (74.6)	27 (62.8)	0.186
BIS-11 (raw), mean (SD) (n=102)	74.25 (16.4)	75.57 (15.9)	72.13 (17.1)	0.304
BGLHA (raw), mean (SD) (n=107)	19.89 (5.9)	20.61 (6.2)	18.93 (5.4)	0.150

Sample sizes differed across clinical scales because scores either marked as “unknown” or “unreported” by the clinician were excluded. Donors with and without CTE were compared using independent samples t-tests for continuous measures, and chi-square or Fisher’s Exact Test for binary measures.

Abbreviations: BRIEF-A MI: Behavioral Rating Inventory of Executive Function Meta-Cognition Index. BRIEF-A BRI: Behavioral Rating Inventory of Executive Function Behavioral Regulation Index. CDS: Cognitive Difficulties Scale. FAQ: Functional Assessment Questionnaire. AES: Apathy Evaluation Scale. GDS: Geriatric Depression Scale. BIS: Barratt Impulsiveness Scale. BGLHA: Brown-Goodwin Assessment for Lifetime History of Aggression.

© 2023 McKee AC et al. *JAMA Neurology*.

eFigure. Multiplex Immunofluorescent Labelling of the CTE Pathognomonic Lesion

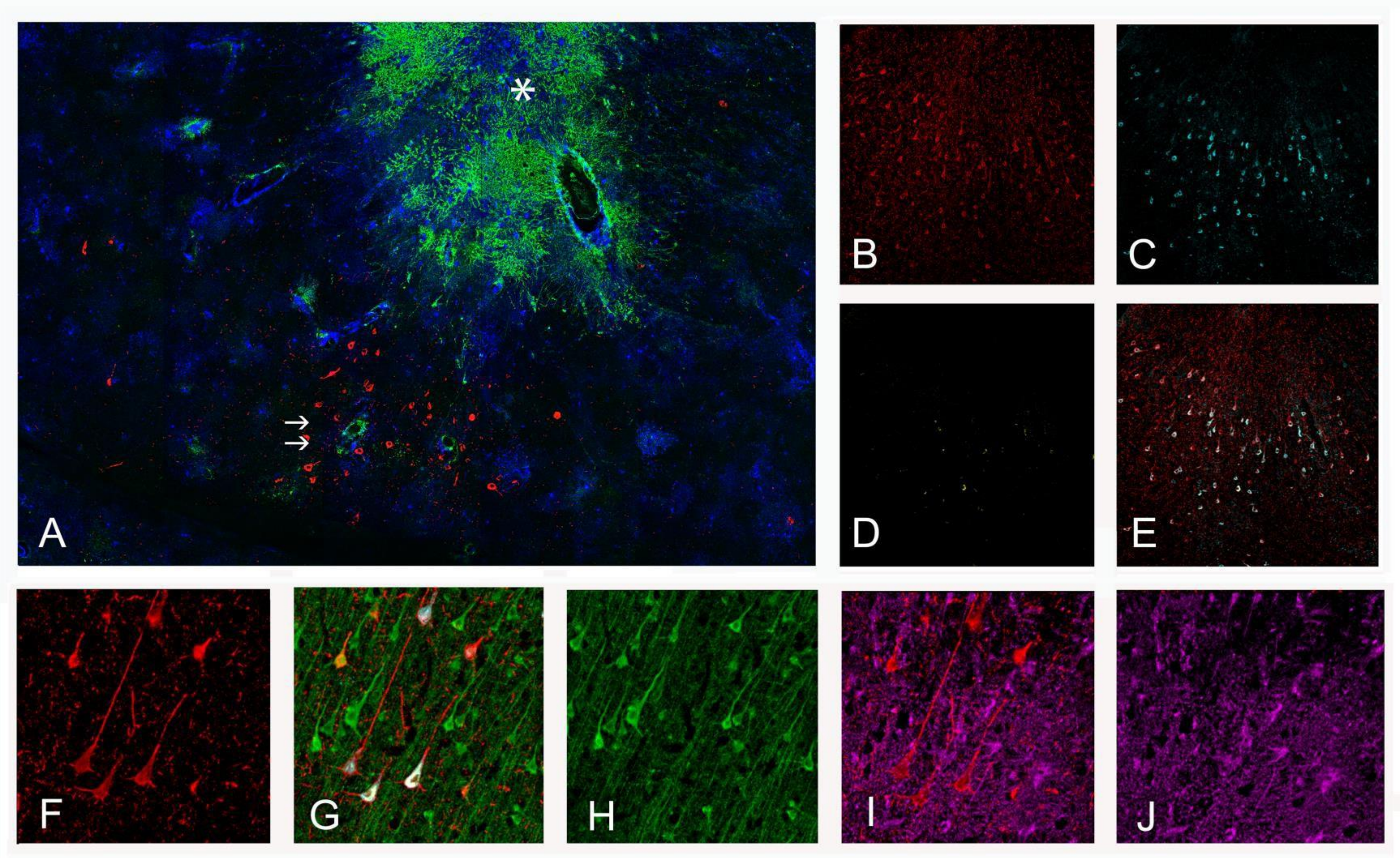

A. Multiplex immunofluorescent labelling of the pathognomonic lesion of CTE (white arrows), a perivascular accumulation of neurofibrillary tangles (NFTs) and dotlike neurites at the depth of the dorsolateral frontal sulcus (asterisk) of a 27-year-old football player with stage III CTE. AT8 (red), aquaporin 4 (green), GFAP (blue). Magnification x 100.

B.-E.) Immunofluorescent labelling of dorsolateral frontal cortex of a 27-year-old football player with stage III CTE stained for (B) AT8, (C) 4R tau, (D) 3R tau, and (E) merged, showing extensive neuronal p-tau deposition and co-localization of AT8 predominantly with 4R tau. Magnification x 100.

F.-J.) Multiplex immunofluorescent labelling of a CTE lesion: (F) AT8 (red), (G) merged AT8, MAP2 (green), 4R (blue) and 3R (yellow), (H) MAP2 (green), (I) AT8 (red) and GFAP (pink), and (J) GFAP (pink) labelling, indicating that neurofibrillary tangles (NFT)-containing cells co-localize with MAP2, a neuronal marker, and not GFAP, a marker of astrocytes.

© 2023 McKee AC et al. *JAMA Neurology*.
