# Supplementary material for: Neuropathologic and Clinical Findings in Young Contact Sport Athletes Exposed to Repetitive Head Impacts
Source: JAMA Neurol. 2023 Aug 28;80(10):1037–50. doi: 10.1001/jamaneurol.2023.2907 (PMC10463175; doi:10.1001/jamaneurol.2023.2907)
Supplement: Supplement 2. — Data Sharing Statement [file jamaneurol-e232907-s002.pdf]

Data Sharing Statement

McKee. Neuropathologic and Clinical Findings in Young Contact Sport Athletes Exposed to Repetitive Head Impacts. *JAMA Neurol*. Published August 28, 2023.
doi:10.1001/jamaneurol.2023.2907

Data
Data available: Yes
Data types: Data dictionary
How to access data: amckee@bu.edu
When available: With publication

Supporting Documents
Document types: None

Additional Information
Who can access the data: anyone requesting the data
Types of analyses: anyone requesting the data
Mechanisms of data availability: with a signed data access agreement
